# The Role of Rifampin in Prosthetic Joint Infections: Efficacy, Challenges, and Clinical Evidence

**DOI:** 10.3390/antibiotics13121223

**Published:** 2024-12-17

**Authors:** Jakrapun Pupaibool

**Affiliations:** Division of Infectious Diseases, Department of Internal Medicine, University of Utah, Salt Lake City, UT 84112, USA; jakrapun.pupaibool@hsc.utah.edu

**Keywords:** rifampin, rifampicin, prosthetic joint infection, periprosthetic joint infection, PJI, biofilm

## Abstract

Rifampin is a crucial antibiotic in the management of prosthetic joint infections (PJI), particularly due to its effectiveness against staphylococcal bacteria and its ability to penetrate and disrupt biofilms. This review evaluates rifampin’s role by examining its mechanism of action, clinical efficacy, and integration into treatment regimens based on recent evidence and guidelines. Rifampin’s synergistic effects with other antibiotics, such as β-lactams and vancomycin, enhance bacterial eradication, and some evidence shows that it improves patient outcomes. However, evidence supporting its use is limited by the scarcity of robust human clinical trials, and challenges such as potential drug interactions and resistance development necessitate careful management. Ongoing research is needed to refine its use and address existing limitations in clinical practice.

## 1. Introduction

Prosthetic joint infections (PJIs) remain one of the most challenging complications following joint arthroplasties, often leading to significant morbidity, increased healthcare costs, and the need for revision surgeries. As antibiotic resistance continues to rise, the choice of antimicrobial agents is critical in managing these infections. Rifampin, also known as rifampicin, is a well-known antibiotic primarily used to treat tuberculosis. It has garnered attention for its unique pharmacokinetic properties and ability to penetrate biofilms, making it a potentially valuable option in the treatment of PJIs [1,2]. Despite its potential, the optimal use of rifampin in this setting remains controversial due to various challenges, including resistance development, drug interactions, and variability in clinical application.

This narrative review explores the multifaceted roles of rifampin in managing PJIs, focusing on its efficacy, the challenges associated with its use, and the current clinical evidence supporting its application. Additionally, this review explores factors influencing rifampin’s effectiveness, such as biofilm penetration, resistance patterns, and combination therapy strategies. By synthesizing the available literature, this review aims to bridge critical knowledge gaps, offering clinicians practical insights to enhance decision making and improve patient outcomes in the management of PJIs. Additionally, the review explores factors influencing rifampin’s effectiveness, such as biofilm penetration, resistance patterns, and combination therapy strategies. By synthesizing the available literature, this review aims to provide a comprehensive understanding of rifampin’s position within the therapeutic landscape of PJIs, ultimately guiding clinicians in making informed decisions for optimal patient outcomes.

## 2. Pharmacology of Rifampin

### 2.1. Pharmacokinetics

Rifampin is well-absorbed following oral administration, with peak plasma concentrations typically achieved within 2 to 4 h. The bioavailability of rifampin ranges from 60% to 80%, influenced by factors such as food intake and gastrointestinal pH [3]. Absorption of rifampin is reduced by about 30% when the drug is ingested with food. To optimize absorption, it is often recommended to take rifampin on an empty stomach [3,4]. It is widely distributed throughout the body and is highly protein-bound. It has a large volume of distribution (approximately 0.8 to 1.6 L/kg) [5], which allows it to penetrate various tissues, including bone and joint, making it particularly relevant for treating infections in prosthetic joints [6].

### 2.2. Drug Interactions

Rifampin undergoes extensive hepatic metabolism, primarily via the cytochrome P450 enzyme system, including isoenzymes CYP2B6, CYP2C8, CYP2C9, CYP2C19, CYP3A4, CYP3A5, and CYP3A7 [7]. This metabolism results in the formation of active and inactive metabolites. It is known to induce several cytochrome P450 enzymes, which can lead to drug interactions with other medications metabolized by this pathway. For orthopedic patients with PJI, who often require anticoagulation with warfarin or direct oral anticoagulants (DOACs) such as apixaban or rivaroxaban [8], the potential for drug–drug interactions with rifampin is a critical concern that must be addressed in their treatment planning. Rifampin can also reduce the effectiveness of oral contraceptives [9] and corticosteroids [10] when taken together. It does so by inducing liver enzymes, which accelerate the metabolism of these drugs, resulting in lower plasma concentrations. For oral contraceptives, this increased clearance can lead to a higher risk of unintended pregnancy, as the contraceptive’s effectiveness is diminished. Similarly, rifampin can decrease the levels of corticosteroids, potentially reducing their anti-inflammatory and immunosuppressive effects. Because of these interactions, it is important to consider alternative methods of contraception or adjust corticosteroid doses when using rifampin. Details on how rifampin affects the levels of specific antimicrobial agents will be discussed in a separate section.

### 2.3. Metabolism and Dose Adjustment

Rifampin generally does not require dose adjustments in patients with renal impairment because it is primarily metabolized in the liver rather than excreted unchanged by the kidneys. In cases of hemodialysis, rifampin is not effectively removed; thus, no additional dosing is necessary post-dialysis. Despite the lack of significant renal elimination, dosing and therapy should still be carefully managed, particularly in combination with other drugs that may require renal dose adjustments or pose additional risks to renal function. Rifampin is primarily excreted in the bile. A significant portion of the drug is eliminated unchanged in the feces, while approximately 30% is excreted in urine as both unchanged drug and metabolites [3,11].

### 2.4. Adverse Effects and Tolerability

Rifampin, while effective, is associated with several side effects that warrant careful consideration in clinical use. Common adverse effects include gastrointestinal disturbances such as nausea, vomiting, and abdominal discomfort, which can impact patient adherence to therapy. Rifampin is also known to cause hepatotoxicity, manifesting as elevated liver enzymes and, in severe cases, liver failure, particularly in patients with pre-existing liver disease or when used in combination with other hepatotoxic drugs. A hallmark side effect of rifampin is its ability to discolor bodily fluids, such as urine, sweat, and tears, to an orange-red hue, which, while harmless, can alarm patients if not forewarned. Hypersensitivity reactions, including rash and fever, are also reported. These side effects underscore the need for vigilant monitoring and patient education when using rifampin in therapeutic regimens [12].

### 2.5. Mechanisms of Action

Rifampin is a broad-spectrum antimicrobial agent effective against certain Gram-positive and Gram-negative bacteria. It is always used in combination with other antibiotics to treat a range of infections, particularly those caused by intracellular bacteria, such as tuberculosis, leprosy, brucellosis, and cat scratch disease. Its mechanism of action disrupts nucleic acid synthesis by inhibiting DNA-dependent RNA polymerase, enabling it to target dormant bacteria with very low division rates. Rifampin effectively penetrates staphylococcal biofilms both in vivo and in vitro [1,2]. However, clinical studies present conflicting results regarding the benefits of rifampin in implant-associated infections.

Biofilm plays a significant role in the persistence of infections associated with implantable devices and prosthetic joints, as it shields organisms from the host’s immune response and enhances their resistance to antimicrobial agents. Key organisms that can form biofilms include Gram-positive and Gram-negative bacteria such as *Staphylococcus aureus* (*S. aureus*), *Staphylococcus epidermidis* (*S. epidermidis*), viridians streptococci, *Enterococcus faecalis*, *Pseudomonas aeruginosa*, *Escherichia coli*, *Klebsiella pneumoniae*, and *Proteus mirabilis* [13]. Additionally, fungi such as *Candida* species can also form biofilms on medical devices [14]. This creates a challenging treatment scenario, as the biofilm’s structure can impede drug penetration and allow for the survival of bacteria even in the presence of antimicrobial agents. Furthermore, the slow growth rate of bacteria within biofilms makes them less susceptible to conventional treatments, complicating infection resolution. As a result, managing infections related to biofilms often requires more aggressive and prolonged therapeutic strategies.

## 3. Dosage of Rifampin in the Context of Prosthetic Joint Infection

Various regimens for daily rifampin administration have been reported, with doses ranging from 600 mg per day [15,16] to 900 mg per day [17,18], either given as twice-daily doses or as a single daily dose. The data indicated that doses higher than 600 mg/day (or more than 10 mg/kg/day) were not well tolerated and did not lead to improved patient outcomes [19,20]. The pharmacokinetic–pharmacodynamic (PK/PD) data for rifampin in tuberculosis (TB) have consistently shown that achieving an optimal area under the curve (AUC) over minimal inhibitory concentration (MIC) (AUC/MIC) ratio is crucial for the drug’s efficacy, prevention of emerging antimicrobial resistance, and promoting the post-antibiotic effect (PAE) [21,22,23,24]. Although direct PK/PD data for rifampin in staphylococcal infections in humans are limited, it is reasonable to extrapolate that the principles underlying the AUC/MIC ratio for TB would similarly apply to staphylococci, given that rifampin’s mechanism of action is based on prolonged drug exposure to disrupt bacterial growth and biofilm formation. As a result, some clinicians have proposed that a single daily dose of rifampin (600 mg once daily) may be as effective as divided doses (300 mg twice daily) [25]. The rifampin dose of 300 mg achieves a peak concentration of 4.0 µg/mL, with a trough level greater than 0.25 µg/mL at 12 h. Given the MIC values for *S. aureus* (0.008–0.15 µg/mL) and *S. epidermidis* (0.004–0.15 µg/mL), both the 300 mg twice-daily and 600 mg once-daily dosing regimens are considered adequate [26].

## 4. Rifampin-Resistant *Staphylococcus* Species

Rifampin is a potent antibiotic against staphylococci; however, it should always be used in conjunction with other antimicrobials, as resistance can develop rapidly when it is used alone. Prolonged use of rifampin or its use as monotherapy can lead to the emergence of rifampin-resistant *S. aureus* [27]. Resistance in both *S. aureus* and *S. epidermidis* is often linked to point mutations in the *rpoB* gene, which encodes the β-subunit of bacterial RNA polymerase, a key target for rifampin’s action [28]. Interestingly, rifampin-resistant *S. aureus* exhibits a growth advantage in aging biofilms but not in liquid (planktonic) cultures [29]. Several studies have highlighted a concerning correlation between specific *rpoB* mutations and decreased susceptibility to other anti-staphylococcal agents such as vancomycin, daptomycin, β-lactams, and imipenem [30,31,32,33]. Additionally, treatment failure is more likely to occur if rifampin is initiated within five days following surgical debridement [34].

## 5. Efficacy of Rifampin in Treating Gram-Positive Prosthetic Joint Infections: Available Evidence

### 5.1. Rifampin for Staphylococcal PJI

Rifampin has been the subject of several clinical investigations, primarily retrospective studies focusing on debridement, antibiotics, and implant retention (DAIR). Evidence supporting its effectiveness in both single-stage and two-stage revision arthroplasty is quite limited. To date, only three randomized controlled trials (RCTs) [18,35,36] have addressed this issue; however, all of them had certain limitations, and two were small-scale studies (Table 1).

The RCT conducted by Zimmerli et al. was the first to evaluate the efficacy of rifampin in combination with other antimicrobial agents for treating staphylococcal PJI involving the hip, knee, and orthopedic implants [18]. The study, published in 1998, assessed the effectiveness of rifampin in treating 33 patients with staphylococcal PJI of the hip and knee and orthopedic implants. The antimicrobial regimen consisted of a two-week course of intravenous flucloxacillin or vancomycin combined with either rifampin or a placebo, followed by a treatment phase of ciprofloxacin paired with rifampin or a placebo. The study reported a cure rate of 100% (12 out of 12) in the ciprofloxacin-rifampin group, compared to 58% (7 out of 12) in the ciprofloxacin-placebo group (*p* = 0.02). Notably, all *S. aureus* strains in this trial were methicillin-susceptible (MSSA). The dose of rifampin used was 450 mg twice daily.

In an open-label, phase-2 RCT, Pushkin et al. evaluated the effectiveness of fusidic acid and rifampin compared to standard-of-care intravenous antibiotics in treating PJI of the hip or knee in 14 participants [36]. Pharmacokinetic assessments revealed that fusidic acid levels were significantly lower than expected across all subjects. This reduction raised concerns about the potential for rifampin-resistant infections and the risk of treatment failure. Consequently, the study was terminated due to the identified drug–drug interaction between fusidic acid and rifampin, which could adversely affect patient outcomes.

The most recent RCT by Karlsen et al., published in 2020, included 99 patients undergoing DAIR for PJI, with 48 patients included in the final analyses [35]. At the two-year follow-up, there was no statistically significant difference in the success rates of eradicating PJI between the treatment groups, with success rates of 74% (17 out of 23) for the rifampin combination therapy group versus 72% (18 out of 25) for the monotherapy group (*p* = 0.88). The study faced challenges, including a high dropout rate, and did not utilize other effective antimicrobial agents with good bone penetration, such as fluoroquinolones and trimethoprim-sulfamethoxazole. High-dose rifampin (900 mg daily) was used in conjunction with oral cloxacillin or vancomycin, and no cases of methicillin-resistant *S. aureus* (MRSA) were reported in this study.

Generally, the retrospective analyses have indicated at least some benefit of rifampin in managing staphylococcal PJI following DAIR procedures [34,37,38,39]. A meta-analysis encompassing 22 relevant studies, predominantly retrospective in nature, examined the role of rifampin in the treatment of PJI. Among the studies that exclusively utilized DAIR (a total of 1427 patients), rifampin did not show a significant protective effect against treatment failure, with an odds ratio (OR) of 0.66 and a 95% confidence interval (CI) of 0.43–1.02. Conversely, studies employing DAIR selectively (a total of 1103 patients) demonstrated a significant reduction in failure rates associated with rifampin, yielding an OR of 0.47 with a 95% CI of 0.27–0.80. Overall, this meta-analysis of 22 studies indicated that incorporating rifampin into standard care significantly reduced failure rates (OR 0.57, 95% CI 0.41–0.78). These findings suggest that while rifampin may offer benefits in treating PJI, further high-quality evidence is necessary to determine its optimal use and dosing across different treatment scenarios [40].

### 5.2. Rifampin for Non-Staphylococcal PJI

Rifampin may have a significant role in combination antimicrobial therapy for treating PJI caused by *Streptococcus* species and *Cutibacterium* (formerly *Propionibacterium*) species, both of which are known for their ability to form biofilms [41,42,43,44]. Four retrospective studies evaluated rifampin’s effectiveness in streptococcal PJIs, all indicating a trend toward improved outcomes [45,46,47]; however, only one study achieved statistical significance (Table 2) [48]. Similarly, two retrospective studies suggested a potential benefit of adding rifampin to the treatment of *Cutibacterium*-associated PJIs in the shoulder, hip, and knee, showing promising success rates [49,50]. Nevertheless, the necessity of incorporating rifampin into the treatment regimen for these infections remains uncertain. Further research is essential to clarify whether the addition of rifampin is advisable for PJIs caused by *Streptococcus* and *Cutibacterium* species.

Due to the limitations of the existing clinical evidence and the absence of good-quality RCTs, the role of rifampin as an adjunctive therapy for PJI caused by Gram-positive bacteria is still debatable.

### 5.3. Combination Therapy and Challenges in the Use of Rifampin in Prosthetic Joint Infection

The strongest evidence supporting rifampin combination therapy lies in its use alongside fluoroquinolones, vancomycin, and beta-lactams. However, it is important to note that rifampin can decrease the plasma levels of several other antibiotics frequently employed in the management of PJIs, such as doxycycline [51], linezolid [52], trimethoprim-sulfamethoxazole (TMP-SMX) [53], clindamycin [54,55,56], and fusidic acid [36], potentially reducing their therapeutic efficacy (Table 3). Rifampin can reduce the plasma levels of azole antifungal agents (such as ketoconazole, itraconazole, and fluconazole) when used concomitantly. This occurs because rifampin is a potent inducer of cytochrome P450 enzymes, particularly CYP3A4, which plays a key role in the metabolism of many azoles. By increasing the activity of these enzymes, rifampin accelerates the breakdown of azole drugs, leading to lower drug concentrations in the bloodstream. As a result, the antifungal effectiveness of azoles may be diminished, potentially compromising the treatment of fungal infections.

Interestingly, while interactions between rifampin and other antibiotics have been documented, these interactions have not consistently translated into clinical failure, as seen in many studies. In studies involving TB treatment, the addition of TMP-SMX to rifampin has been shown to have a synergistic effect [57] and prevent the emergence of drug-resistant *Mycobacterium tuberculosis* in vitro [58]. The combination of rifampin and doxycycline is widely recommended for treating brucellosis. A study indicated that ofloxacin-rifampicin combination therapy offers benefits such as shorter treatment duration and quicker resolution of fever while maintaining comparable efficacy to the doxycycline-rifampicin regimen in managing brucellosis [59]. Additionally, triple antibiotic therapy consisting of doxycycline, TMP-SMX, and rifampicin has proven effective in treating brucellosis spondylitis [60], while both doxycycline and TMP-SMX have been demonstrated to reduce their levels when used with rifampicin in other studies. A study using a mouse model indicated that linezolid–rifampin combination therapy was as effective as intravenous antibiotic–rifampin combinations for treating PJI [61].

Clinical evidence comparing various regimens of rifampin combination therapy for PJI in humans is limited. An observational study found that combining rifampin with antibiotics other than fluoroquinolones or clindamycin was associated with a higher rate of treatment failure [34]. Conversely, another retrospective study indicated that rifampin combined with linezolid, TMP-SMX, or clindamycin resulted in higher failure rates compared to combinations with fluoroquinolones or amoxicillin, as well as higher failure rates compared to linezolid or TMP-SMX monotherapy [62].

The combination of rifampin with newer antimicrobial agents, such as daptomycin, oritavancin, and dalbavancin, offers promising therapeutic potential for managing complex infections. Rifampin and daptomycin have shown enhanced bactericidal activity in treating osteomyelitis from MRSA [63]. Similarly, rifampin combined with oritavancin or dalbavancin has demonstrated in vitro synergy and improved efficacy in limited studies [64,65]. However, evidence supporting these combinations in the context of PJI remains lacking. Further large-scale clinical trials are necessary to confirm their safety, efficacy, and optimal application in various infection types, including PJI.

These data suggest that, despite the potential for altered drug levels, the clinical significance of these interactions may vary, warranting further investigation to better understand the implications for treatment strategies in PJI. As such, clinicians should exercise caution when prescribing rifampin in conjunction with other antimicrobial agents, as the potential for altered drug levels could impact treatment outcomes. Further research is needed to elucidate these interactions and provide clearer guidance on the safe and effective use of rifampin in combination therapy, specifically for PJIs. Overall, careful consideration of drug interactions is crucial for optimizing patient management and achieving the best possible results in the treatment of these complex infections.

## 6. Clinical Significance and Recommendations

The findings of this review underscore rifampin’s potential as a cornerstone in the management of PJI, particularly those caused by *Staphylococcus* species. Its biofilm-penetrating ability uniquely positions rifampin as a key adjunct in combination therapy, enhancing the efficacy of other antibiotics against biofilm-associated bacteria. However, its use comes with critical considerations that must be addressed to maximize its benefits and minimize risks.

Combination therapy is recommended to reduce the risk of resistance development, with rifampin commonly paired with agents like fluoroquinolones, vancomycin, and beta-lactams. It is crucial to initiate rifampin only after adequate surgical debridement and at least five days after the surgical procedure, as premature use without proper source control may lead to suboptimal outcomes and increased resistance risk.

To mitigate resistance, susceptibility testing should be performed before initiating rifampin therapy. Clinicians should avoid its use in infections with rifampin-resistant organisms. The potential for drug–drug interactions must also be carefully managed, particularly with anticoagulants, immunosuppressants, and oral contraceptives. Close monitoring of liver function is essential, especially in patients with pre-existing liver conditions or those receiving other hepatotoxic drugs. Educating patients about common side effects, such as the discoloration of bodily fluids, can help maintain adherence to treatment. Treatment plans should be tailored to the individual patient, considering factors such as comorbidities, surgical intervention types, and the infection’s microbiological profile.

## 7. Future Directions

Future research should focus on several key areas to enhance our understanding and application of rifampin in the management of PJI. First, larger-scale randomized controlled trials are necessary to provide more definitive evidence regarding the efficacy of rifampin, particularly in combination therapy with other antimicrobial agents. Investigating the optimal dosing regimens and treatment durations for rifampin in various contexts could further refine clinical practices. Additionally, understanding the impact of rifampin on biofilm formation and maintenance could provide insights into novel therapeutic approaches, including potential adjunctive therapies aimed at disrupting biofilms. Lastly, investigating the pharmacokinetic interactions between rifampin and other commonly used antimicrobial agents in orthopedic infections will help in designing safer and more effective combination therapies. This could ultimately lead to improved outcomes for patients with PJI, particularly as antibiotic resistance continues to challenge current treatment paradigms.

## 8. Conclusions

In conclusion, rifampin holds significant potential in the management of PJIs, particularly those caused by biofilm-producing organisms, especially *Staphylococcus* species. Current evidence supports its use in combination therapies to enhance efficacy and improve outcomes. However, challenges such as resistance development, hepatotoxicity, and drug interactions necessitate a cautious, individualized approach in clinical practice. To maximize its therapeutic benefits, further high-quality research is needed to define optimal dosing strategies, timing, and combination regimens. By addressing these gaps, rifampin can be more effectively integrated into comprehensive treatment strategies, ultimately reducing the burden of PJIs and improving patient care.

## Figures and Tables

**Table 1:** Summary of Randomized Controlled Trials Assessing the Efficacy of Rifampin in the Treatment of Staphylococcal Prosthetic Joint Infections.

Reference	First Author	Year of Publication	Types of Arthroplasty Revision	Sample Size	Cure Rate (Rifampin vs. Control)
[18]	Zimmerli W	1998	DAIR	33	100% vs. 58%
[36]	Pushkin R	2016	DAIR, 2-stage exchange	14	57% vs. 57%
[35]	Karlsen E	2020	DAIR	99	74% vs. 72%

**Table 2:** Summary of Retrospective Studies Assessing the Efficacy of Rifampin in the Treatment of Streptococcal Prosthetic Joint Infections.

Reference	First Author	Year of Publication	Types of Arthroplasty Revision	Sample Size	Cure Rate (Rifampin vs. Control)
[45]	Andronic O	2021	mixed	22	80% vs. 65%
[46]	Mahieu R	2019	mixed	95	74% vs. 72%
[47]	Wouthuyzen-Bakkler M	2019	DAIR	14	77% vs. 58%
[48]	Faux E	2009	DAIR	95	85% vs. 42%

**Table 3:** Antimicrobial Agents That Have Evidence of Reduced Plasma Levels When Used Concomitantly with Rifampin for the Treatment of Periprosthetic Joint Infection (PJI).

Antimicrobial Agents	Drug Level	Study Population
Doxycycline	Decrease	Humans with brucellosis
Clindamycin	Decrease	Humans with osteoarticular infections
Linezolid	Decrease	Healthy mice
Trimethoprim-sulfamethoxazole	Decrease	Humans with HIV infection
Fusidic acid	Decrease	Humans with PJI
